# Quantitative phase imaging reveals matrix stiffness-dependent growth and migration of cancer cells

**DOI:** 10.1038/s41598-018-36551-5

**Published:** 2019-01-22

**Authors:** Yanfen Li, Michael J. Fanous, Kristopher A. Kilian, Gabriel Popescu

**Affiliations:** 1 Department of Bioengineering, University of Illinois at Urbana-Champaign, Urbana, Illinois 61801 USA; 2 Quantitative Light Imaging Laboratory, Department of Electrical and Computer Engineering, Beckman Institute for Advanced Science and Technology, University of Illinois at Urbana-Champaign, Urbana, Illinois 61801 USA; 3 School of Chemistry, School of Materials Science and Engineering, Australian Centre for NanoMedicine, University of New South Wales, Sydney, NSW 2052 Australia

## Abstract

Cancer progression involves complex signals within the tumor microenvironment that orchestrate proliferation and invasive processes. The mechanical properties of the extracellular matrix (ECM) within this microenvironment has been demonstrated to influence growth and the migratory phenotype that precedes invasion. Here we present the integration of a label-free quantitative phase imaging technique, spatial light interference microscopy (SLIM)—with protein-conjugated hydrogel substrates—to explore how the stiffness of the ECM influences melanoma cells of varying metastatic potential. Melanoma cells of high metastatic potential demonstrate increased growth and velocity characteristics relative to cells of low metastatic potential. Cell velocity in the highly metastatic population shows a relative insensitivity to matrix stiffness suggesting adoption of migratory routines that are independent of mechanics to facilitate invasion. The use of SLIM and engineered substrates provides a new approach to characterize the invasive properties of live cells as a function of microenvironment parameters. This work provides fundamental insight into the relationship between growth, migration and metastatic potential, and provides a new tool for profiling cancer cells for clinical grading and development of patient-specific therapeutic regimens.

## Introduction

The mechanical properties of the tumor microenvironment plays a role in guiding cancer development, transformation and invasive processes^1^. The extracellular matrix (ECM) is an important component of the microenvironment and consists of proteins, glycoproteins, proteoglycans, polysaccharides, and other biochemically distinct components^2,3^. This ordered structure contains unique chemical, physical, and mechanical properties which are essential in numerous physiological processes including homeostasis^4^, differentiation^5,6^ and migration^7,8^. The ECM proteins also bind to soluble growth factors to regulate their activation and distribution in order to pass signals into the cell^9^. The biomechanical properties of the ECM, such as its viscoelasticity, can also influence disease development and progression^10,11^.

The ECM is a dynamic system that is constantly being remodeled by the cells that inhabit it. This in turn influences adjacent cells to modify their behavior^12^. In the tumor microenvironment, abnormal ECM dynamics are common and contribute to the process of progression, transformation, and dissemination. For instance, a hallmark of cancer is the excess production of ECM proteins including collagen I, II, III, V, and IX, which leads to tissue fibrosis^13–17^. This in turn increases the stiffness of the tumor microenvironment as compared to the surrounding tissue, which then further enhances cancer progression via reducing levels of tumor suppressors PTEN and HOXA9 in cancer cells^17,18^. Weaver and colleagues demonstrated how breast adenocarcinoma cells will secrete lysyl oxidase which crosslinks ECM proteins, leading to additional stiffening to facilitate invasion^19^. This increase in stiffness also impacts surrounding cells including creation of cancer-associated fibroblasts^20^ and tumor-activated macrophages^21^.

Cancer metastasis is a multistep process which involves the intravasation from the tumor, survival in the circulatory and/or lymphatic system, extravasation and colonization at a distant site^22,23^. In order to intravasate or extravasate from solid tumors, cancer cells will generally undergo transformations between epithelial phenotypes and invasive mesenchymal or amoeboid phenotypes^24^. Several groups have identified key roles for the ECM in facilitating these transformations, including a pronounced role for the mechanics of the surrounding matrix^25^. For example, during epithelial to mesenchymal transition (EMT) where polarized epithelial cells transition to more mobile mesenchymal cells during biological processes such as embryogenesis and cancer progression^24^, laminin-rich ECM can suppress EMT, whereas fibronectin-rich ECM can promote it^26^. Stiffening of the microenvironment has also been shown to drive EMT of breast tumor cells, increasing its invasion potential and metastasis^27^ and tissue polarity aids death resistance of mammary tumor cells^28^. In histopathology, recent work has revealed that tumor microenvironment carries prognosis information^29–33^. While considerable work has led to the identification of processes underlying cancer cell invasiveness, no technique can simultaneously probe the interdependence of matrix parameters on multiple complex functions, i.e. migration and growth, which are critical aspects of invasion.

**Figure 1:**
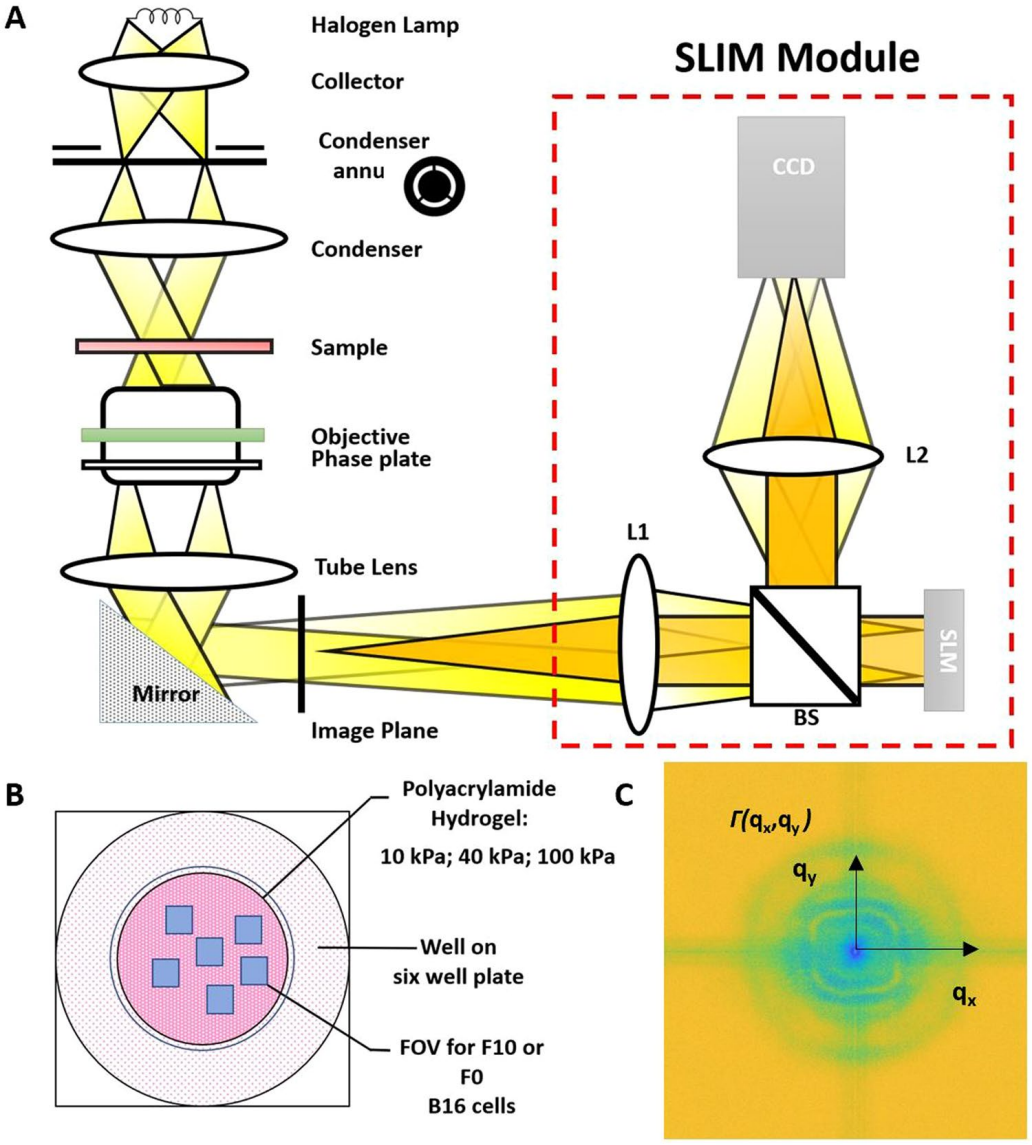
Schematic setup for SLIM. (**A**) The SLIM module is attached to a commercial phase contrast microscope (Axio Observer Z1, Zeiss). (**B**) Experimental well plate setup: 10 kPa, 40 kPa and 100 kPa polyacrylamide hydrogels were prepared in different wells. (**C**) Decay rate vs. spatial mode associated with phase images generated with (**A**).

In this paper, we use spatial light interference microscopy (SLIM) as a label-free quantitative phase imaging (QPI)^34^ technique to explore how matrix stiffness influences cancer cell growth and migration in real time (Fig. 1A). Quantitative phase imaging is a method that can measure nanometer scale pathlength scale changes in a biological specimen. Typical quantitative phase methods, however, use coherent light sources that compromise the contrast of the images with speckles. SLIM overcomes this drawback with the use of a broadband field, and measures nanoscale details and dynamics in live cells via interferometry^35^. SLIM couples Zernike’s phase contrast microscope, which produces high contrast images of transparent samples, with Gabor’s holography, which records the sample’s phase information. The result is a quantitative optical pathlength map across the specimen. Here we use malignant melanoma as a model metastatic cancer—with subclones of varying metastatic potency, including a putative cancer stem cell isolated through matrix engineering^36,37^. The B16 melanoma cells are ideal as model cancer cell lines when studying metastasis due to the same parental tumor background with different degrees of metastatic potential^38^. We show that metastatic potential is underpinned by specific growth and migratory characteristics that are dependent on the stiffness of the matrix.

## Methods

Unless otherwise noted, all materials were purchased from Sigma-Aldrich. Tissue culture plastic ware was purchased from VWR. Glass coverslips were purchased from Fisher Scientific. Cell culture media and reagents were purchased from Gibco.

### Cell Culture

B16 F0 and B16 F10 (ATCC), mouse melanoma cells lines were cultured according to the recommended protocols. B16 F0 cells exhibit less metastatic potential and B16 F10 cells have higher metastatic potential. Cells were passaged at ~80% confluency with 0.25% Trypsin:EDTA and media was changed every 3–4 days. For imaging, cells were seeded at ~50,000 cells/cm^2^ in a 6 well glass bottom plate (P06-20-1.5-N) and were imaged for a period of 24 hours at 30-minute intervals and a capture speed of 6 frames/s. The cells were imaged at incubator conditions. Several frames were selected in each well for time-lapse SLIM measurements (Fig. 1B). For immunofluorescence, cells were seeded on patterned polyacrylamide hydrogels at ~50,000 cells/cm^2^ and cultured for 5 days before fixation.

### Immunocytochemistry

B16 F0 and B16 F10 cells on surfaces were fixed with 4% paraformaldehyde (Alfa Aesar) for 20 minutes at room temperature. 0.1% Triton X-100 in PBS was added for 30 minutes to permeabilize cells and blocked with 1% bovine serum albumin (BSA) for 15 minutes. Cells were labeled with mouse anti- α5β1 (1:200 dilution, Emd Millipore) primary antibody in 1% BSA/PBS at 4 °C overnight. Goat 647-anti-mouse (1:200 dilution) along with Hoechst 33342 (1:3000 dilution) was used for secondary labeling and were incubated with cells for 20 minutes in a humid chamber (37 °C). Immunofluorescence microscopy was conducted with a Leica Microsystems DMi8 confocal microscope.

### Gel Preparation

10 kPa, 40 kPa, and 100 kPA polyacryamide hydrogels were fabricated as previously described to simulate the range of stiffnesses found *in vivo*^39^. Briefly, a mixture of 5% polyacrylamide and 0.15% bis-acylamide were created for each desired stiffness which was then reacted with 0.1% Ammonium Persulfate (APS) and 0.1% Tetramethylenediamine (TEMED). Solutions were pipetted onto a hydrophobically treated glass slide (Rain-X) and an aminopropyltriethoxysilane (APTES)-silanized glass coverslip was placed on top to create a sandwich. After polymerization, gels were lifted off of the base coverslip and immersed in 55% hydrazine hydrate (Fisher) for one hours and washed in 5% glacial acetic acid for one hour.

### Gel Patterning

Polydimethysiloxane (PDMS, Polysciences, Inc) was polymerized on top of SU-8 patterned silicon masters fabricated via conventional photolithography to create PDMS stamps. 25 µg/ml fibronectin was incubated with Sodium Periodate for 45 minutes and pooled on top of the patterned PDMS stamps for 30 minutes. Stamps were then dried under air for 30 seconds and applied to the surface of hydrazine treated hydrogels that were dried at room temperature for one hour to form desired patterns.

#### SLIM

Measurements were made using the SLIM system, comprising an inverted phase contrast microscope (Axio Observer Z1, Zeiss, in this case) and an add-on module (CellVista SLIM Pro, Phi Optics, Inc.). SLIM generates quantitative phase images of the sample that informs on its cell dry mass density at femtogram precision^40,41^. Quantitative phase methods typically use coherent light sources that compromise the contrast of the images with speckles. SLIM overcomes this drawback with the use of a broadband field, enabling highly sensitive measurements. SLIM also offers the advantage of imaging cells without any extraneous label, facilitating long-term imaging without inflicting cellular damage.

A) Dry mass: The dry mass surface density (*ρ*) of cellular matter was obtained from SLIM phase images using the following relationship,

1 $$\rho (x,y)=\frac{\lambda }{2\pi \eta }\,\phi (x,y),$$

where *λ* is the center wavelength of the optical source, *η* = 0.2 ml/g, corresponding to an average of reported values, and *φ* is the phase values of the cells. The total dry mass of a cell was computed by integrating *ρ* over all cellular areas and was used to quantify cell growth in a noninvasive fashion^40,42^.

B) Dispersion-relation phase spectroscopy DPS: To study the dynamics of cellular mass transport, we employed the dispersion phase spectroscopy (DPS) method^43–46^. This computational technique enables the extraction of spatiotemporal intracellular mass transport from a series of time-lapse phase images. The dry mass density dynamics is governed by a advection-diffusion equation,

2 $$D{\nabla }^{2}\rho ({\bf{r}},t)-{\bf{v}}\cdot \nabla \rho ({\bf{r}},t)-\frac{\partial }{\partial t}\rho ({\bf{r}},t)=0,$$

where *D* is the average diffusion coefficient and **v** is the advection velocity. The temporal autocorrelation function at each spatial frequency, *q*, with temporal delay, *τ*, is

3 $$g(q,\tau )={e}^{i{{\bf{v}}}_{0}\cdot {\bf{q}}\tau }{e}^{-q{\rm{\Delta }}v\tau -D{q}^{2}\tau },$$

where **v**_0_ is the mean and Δ*v* the standard deviation of the velocity distribution. By taking the azimuthal average of the spatial power spectrum, we obtained the 1D decay rate, the *dispersion relation*, Γ(*q*) = Δ*vq* + *Dq*^2^, (Fig. 1C). Thus, the *Dq*^2^ term contains the random (passive, equilibrium) component of cellular transport, while Δ*vq* the deterministic (active, out-of-equilibrium) one. The relationship between decay rate and spatial frequency was thus used to obtain information about the velocity distribution of mass transport. Since it is calculated over the entire field of view, DPS is highly conducive to automated and high-throughput analysis. And because the calculation is based on whole frame analysis, it generates comprehensive information on cellular distribution on a range of relevant spatial scales.

## Results

### Relationships between the degree of metastatic potency and growth responsivity to matrix stiffness

**Figure 2:**
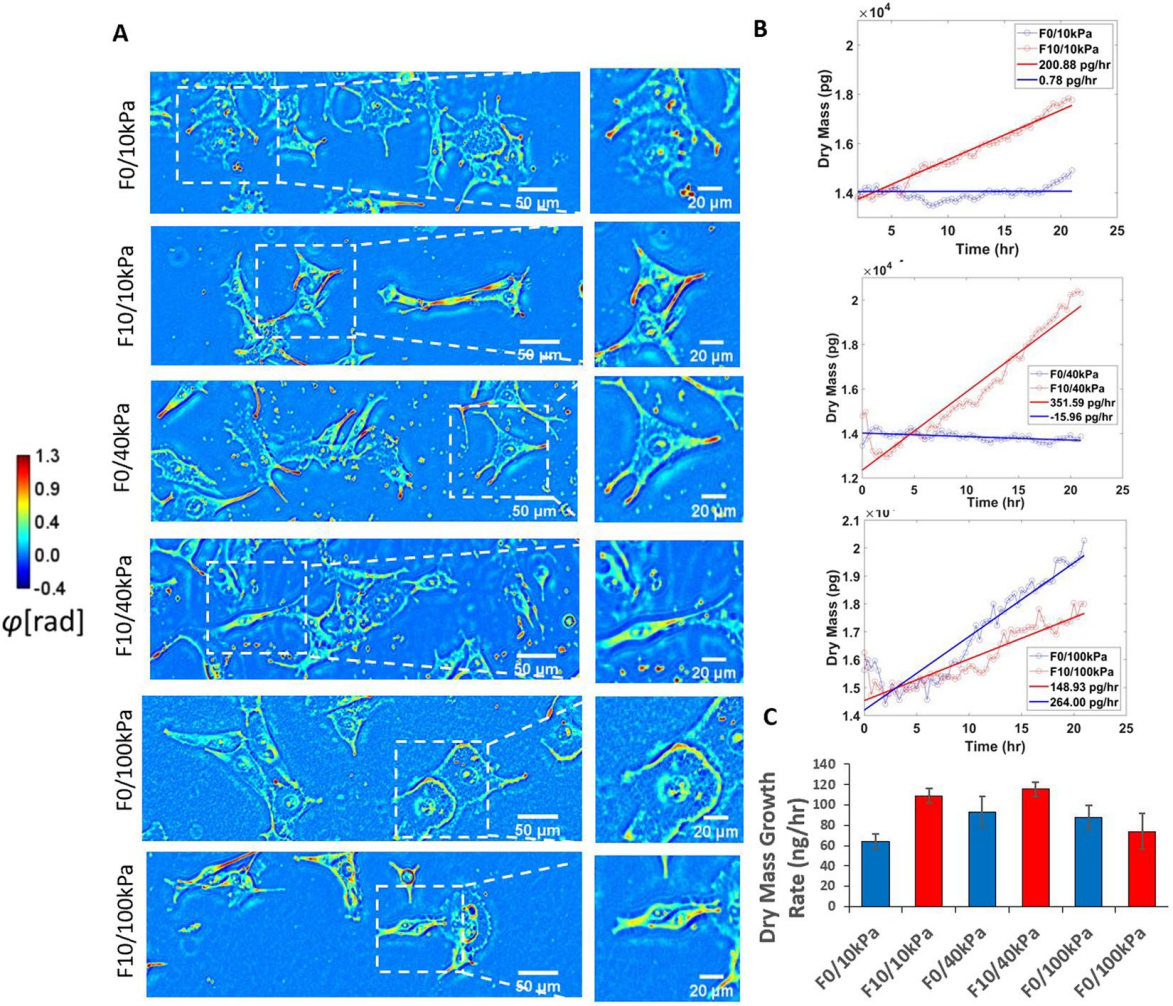
B16 F0 and B16 F10 morphology and dry mass growth profile. (**A**) Phase images produced with SLIM of B16 F0 and B16 F10 on 10 kP, 40 kPa, and 100 kPa polyacrylamide hydrogels indicating example morphology. (**B**) Example dry mass growth profiles of cells. (**C**) Average dry mass growth rate of cells. These results are averaged over 3 experiments, each providing 24 fields of view for analysis. Error bars indicate ±SEM.

To explore how matrix stiffness affects invasiveness of melanoma cancer cells, focusing on fibronectin rich environments, we cultured two types of B16 melanoma cell lines of varying metastatic potential on hydrogel matrices of varying stiffness approximating cancerous tissue and other stiffer sites of common metastasis^47,48^. To do this, we used the well-established material polyacrylamide that can be formulated to span the wide range of all physiologically relevant moduli^49,50^, with a covalent protein conjugation method involving hydrazine activation of acrylamide, oxidation of protein and deposition through contact printing^36,37^. Cells cultured on fibronectin coated polyacrylamide can freely migrate and proliferate with no ill effects within the imaged time period. B16 F0s, cells of lower metastatic potential, and B16 F10s, cells of higher metastatic potential, were seeded on hydrogels and imaged under the SLIM system in order to investigate cellular response upon first contact with a new stiffness. SLIM imaging has the benefit of allowing label-free measurement of cell growth by quantifying the dry mass of the cell instead of overall volume. The dry mass of the cell indicates the amount of total protein within the cell and is a better measurement of cell growth. Previous research has shown that melanoma exhibit higher proliferation at higher stiffness^51^, however previous research often looks at the total volume of cells when comparing growth rates. Cell volume can change in response to external cues such as stiffness due to water efflux^52^. B16 F0 and B16 F10 were seeded onto polyacrylamide hydrogels of 10 kPa, 40 kPa, and 100 kPa and cells attached onto surfaces freely and exhibited healthy morphology (Fig. 2A). Once cells were fully attached, we performed SLIM imaging for 24 hours. The statistical method used to interpret the significance of the results is the student’s *t*-test.

Cellular growth was assessed as aggregate dry mass within a frame, at each time point. Linear regressions were fitted to the changes of dry mass over time (Fig. 2B), and growth rates were determined from their slopes. Dry mass of both cell types increased linearly for 24 hours after attachment (Fig. 2B,C). At lower stiffness of 10 kPa and 40 kPa, the more metastatic B16 F10 had a significantly higher dry mass growth rate of 1092 pg/hr and 1157 pg/hr, respectively, as compared to the less metastatic B16 F0, which had a dry mass growth rate of 641 pg/hr and 930 pg/hr, respectively (p = 0.00086). At the higher stiffness of 100 kPa, B16 F0 had similar dry mass growth to the lower stiffnesses at 880 pg/hr (Fig. S3), whereas B16 F10 decreased to 740 pg/hr (p = 0.27). These data supports previous work which showed that metastatic melanoma cell lines exhibited lower proliferation at a higher stiffness of 2.92 MPa as compared to 0.75 MPa^51^.

### Metastatic cells display similar velocity across matrices of variable stiffness

**Figure 3:**
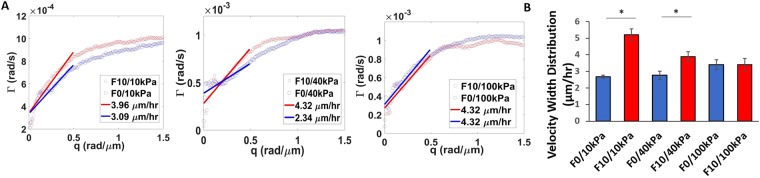
B16 F0 and B16 F10 velocity width distributions on 10 kPa, 40 kPa, and 100 kPa. (**A**) Azimuthal average of decay rates with slopes of linear fits corresponding to the value of the velocity width distribution of cells on 10 kPa, 40 kPa, and 100 kPa. (**B**) Average velocity width distributions for substrates of each stiffness. N = 3. Error bars indicate ± SEM. *p-value < 0.05 (0.027 for 10 kPa and 0.041 for 40 kPa).

As a novel methodology to investigate migration characteristics that may correspond to invasive potential, we calculated velocity distribution width (VDW) for melanoma cells on hydrogels of different stiffness. This metric was obtained from the slope of the decay rate within the spatial frequency range of 0 rad/μm to 0.5 rad/μm, which corresponds to structures from as large as the field of view, down to 6.28 um. The values beyond 0.5 rad/μm do not correspond to total cellular movement, but finer intracellular dynamics. Figure 3A shows example plots of DPS curves corresponding to each grade of stiffness. Curves associated with the highly metastatic B16 F10 cells (red) show steeper slopes (p < 0.05, student’s *t-test*) at lower spatial frequency (*q* values below 0.1) than curves associated with the lowly metastatic B16F0 cells (blue), except for the stiffest substrate condition of 100 kPa. The slopes of these linear fits represent the velocity distribution widths of the cells, which is an indication of their overall transport speed. On average, the more metastatic B16 F10 cells had similar VDW of 2.43 µm/hr, 2.28 µm/hr, and 2.06 µm/hr at 10 kPa, 40 kPa, and 100 kPa, respectively, indicating that stiffness has little influence on B16 F10 migration velocity (Fig. 3B). In contrast, stiffness plays a pronounced role in the migration profile of the less metastatic B16 F0. While B16 F0 had a VWD of 1.74 µm/hr and 1.56 µm/hr at the lower stiffnesses of 10 kPa and 40 kPa, VDW increased to 2.01 µm/hr at 100 kPa. This increase in migration velocity is in line with previous reports that demonstrated relationships between matrix stiffness and the development of migratory invasive phenotypes^53,54^.

### Engineering a tumorigenic phenotype *in vitro* mimics characteristics of high metastatic potential

**Figure 4:**
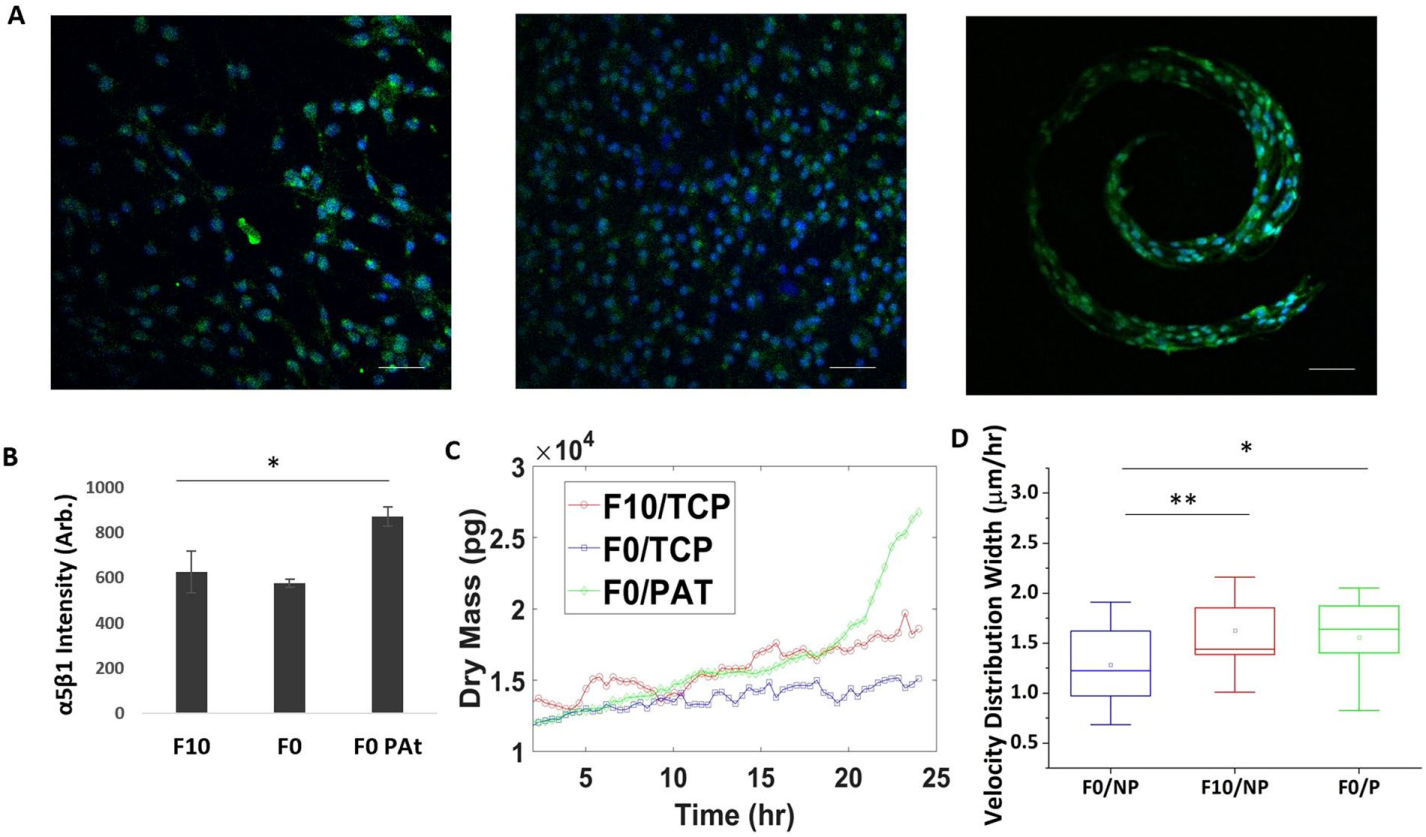
Response of CSC primed B16 F0s to stiffness. (**A**) Immunofluorescence images of α5β1 (green) in patterned B16 F0, non-patterned B16 F0s, and non-patterned B16 F10; nuclei stained with Hoescht (blue). (**B**) Quantification of fluorescence intensity with ImageJ. (**C**) Example of dry mass growth rate of cells (**D**) Average velocity width distribution of cells. N = 3. TCP-tissue culture plastic; PAT - pattern. Error bars indicate ± SEM. Scale bar indicate 50 μm. **p-value < 0.05 (0.034), *p-value < 0.1 (0.058).

A prevailing hypothesis regarding cancer metastasis is the presence of a slow-cycling stem-like cancer cell (herein referred to as CSC) that is primed for invasion and dissemination^55^. The stem fraction in B16 melanoma cells has been engineered through enrichment on fibrin gels^56^ and through control of tumor perimeter topology^36^. To further explore growth and velocity as a function of substrate stiffness and metastatic potential, we cultured B16 F0s in microconfinement for 5 days, an approach that has previously been shown to prime stem-fraction and increase metastatic potency^36^. After removal from microconfined culture and transfer to uniform hydrogels, the microengineered B16 F0s were immunolabeled for the fibronectin-integrin adhesion marker α5β1 which was demonstrated previously to facilitate invasion^36^ (Fig. 4A); micropatterned cells show enhanced adhesion through α5β1 (Fig. 4B). The engineered B16 F0s showed significantly different growth and velocity profiles compared to those cultured on planar gels, with characteristics similar to the highly invasive B16 F10 (Fig. 4C,D). VDW of patterned B16 F0 cells were significantly higher than non-patterned B16 F0 cells, demonstrating velocity characteristics more closely aligned with those of the highly metastatic B16 F10 cells (Fig. 4D). Interestingly, the engineered B16 F0s display non-linear growth characteristics that are more similar to cancer cell growth within a bulk tumor^57^. This finding underscores the versatility of our label-free approach in identifying differences in cell growth rates, and supports the notion that stiffness independent growth and velocity may be a property of cells that are primed for invasion.

## Conclusions

Cancer cell growth and migration are critical aspects underlying oncogenesis, with clear roles during all stages of progression and metastasis. In this paper, we uncover differences in cancer cell behavior as a function of metastatic potential and the mechanics of the underlying matrix through the combination of engineered extracellular matrices and quantitative phase imaging. Cells with higher metastatic potential exhibited greater growth rate than their less metastatic counterpart on soft matrices, and comparable growth rates on stiff matrices. In addition, high metastatic potential corresponds with higher migration profiles, as determined by the velocity width distribution, which was relatively insensitive to changes in stiffness. This is in contrast to the cells of lower metastatic potential, which demonstrated a stiffness dependence in migratory behavior, consistent with previous studies^53,54^. This is important because it suggests that invasive processes underlying metastasis correspond to a cell’s ability to proliferate and migrate irrespective of matrix stiffness. To supplement these results, we primed the cells of lower metastatic potential to a highly aggressive metastatic phenotype through a matrix engineering approach^36^, and demonstrated that these cells adopt characteristics closely aligned with the cells of higher metastatic potential. Interestingly, this stem cell-like population shows non-linear growth characteristics more akin to proliferation of cancer cells within a growing tumor^57^. In addition, this is consistent with recent work demonstrating exponential growth of cancer stem cells when cultured *in vitro*^58^. In conclusion, we have demonstrated how combining quantitative phase imaging with engineered extracellular matrices can reveal changes in growth and velocity during culture that may prove useful as a label free approach to classifying invasiveness and metastatic potential. Future scope includes using these tools to study patient cells from biopsy or resection towards new diagnostic and prognostic assays to guide cancer management and therapeutic intervention.

## Electronic supplementary material

Supplementary Information
