# Author Correction: Quantitative phase imaging reveals matrix stiffness-dependent growth and migration of cancer cells

**DOI:** 10.1038/s41598-020-75941-6

**Published:** 2020-10-29

**Authors:** Yanfen Li, Michael J. Fanous, Kristopher A. Kilian, Gabriel Popescu

**Affiliations:** 1 Department of Bioengineering, University of Illinois at Urbana-Champaign, Urbana, IL 61801 USA; 2 Quantitative Light Imaging Laboratory, Department of Electrical and Computer Engineering, Beckman Institute for Advanced Science and Technology, University of Illinois at Urbana-Champaign, Urbana, IL 61801 USA; 3 School of Chemistry, School of Materials Science and Engineering, Australian Centre for NanoMedicine, University of New South Wales, Sydney, NSW 2052 Australia

Correction to: *Scientific Reports* 10.1038/s41598-018-36551-5, published online 22 January 2019

The original version of this Article contained an error in the Abstract.

“Cell velocity in the highly metastatic population shows a relative insensitivity to matrix stiffness”.

now reads:

“Cell velocity in the highly metastatic population shows a relative stability at higher matrix stiffness”.

Additionally, this Article contained an error in Figure 2, where panel (c) used incorrect units.

Furthermore, this Article contained errors in the Results section.

“At lower stiffness of 10 kPa and 40 kPa, the more metastatic B16 F10 had a significantly higher dry mass growth rate of 1092 pg/hr and 1157 pg/hr, respectively, as compared to the less metastatic B16 F0, which had a dry mass growth rate of 641 pg/hr and 930 pg/hr, respectively (p = 0.00086). At the higher stiffness of 100 kPa, B16 F0 had similar dry mass growth to the lower stiffnesses at 880 pg/hr (Fig. S3), whereas B16 F10 decreased to 740 pg/hr (p = 0.27).”

now reads:

“At lower stiffness of 10 kPa and 40 kPa, the more metastatic B16 F10 had a significantly higher dry mass growth rate of 109.2 pg/hr and 115.7 pg/hr, respectively, as compared to the less metastatic B16 F0, which had a dry mass growth rate of 64.1 pg/hr and 93.0 pg/hr, respectively (p = 0.00086). At the higher stiffness of 100 kPa, B16 F0 had similar dry mass growth to the lower stiffnesses at 88.0 pg/hr (Fig. S3), whereas B16 F10 decreased to 74.0 pg/hr (p = 0.27).”

“On average, the more metastatic B16 F10 cells had similar VDW of 2.43 µm/hr, 2.28 µm/hr, and 2.06 µm/hr at 10 kPa, 40 kPa, and 100 kPa, respectively, indicating that stiffness has little influence on B16 F10 migration velocity (Fig. 3B). In contrast, stiffness plays a pronounced role in the migration profile of the less metastatic B16 F0. While B16 F0 had a VWD of 1.74 µm/hr and 1.56 µm/hr at the lower stiffnesses of 10 kPa and 40 kPa, VDW increased to 2.01 µm/hr at 100 kPa.”

now reads:

“On average, the more metastatic B16 F10 cells had VDWs of 5.2 μm/hr, 4.0 μm/hr, and 3.5 μm/hr at 10 kPa, 40 kPa, and 100 kPa, respectively, indicating that lower stiffness has some influence on B16 F10 migration velocity (Fig. 3B). As well, stiffness plays a role in the migration profile of the less metastatic B16 F0. While B16 F0 had a VWD of 2.5 μm/hr and 3 μm/hr at the lower stiffnesses of 10 kPa and 40 kPa, VDW increased to 3.5 μm/hr at 100 kPa.”

Finally, this Article contained an error in the Conclusion section.

“In addition, high metastatic potential corresponds with higher migration profiles, as determined by the velocity width distribution, which was relatively insensitive to changes in stiffness.”

now reads:

“In addition, high metastatic potential corresponds with higher migration profiles, as determined by the velocity width distribution, which did not increase with increasing stiffness.”

These errors have now been corrected in the PDF and HTML versions of the Article.

