# Supplementary material for: Quantitative phase imaging reveals matrix stiffness-dependent growth and migration of cancer cells
Source: Sci Rep. 2019 Jan 22;9:248. doi: 10.1038/s41598-018-36551-5 (PMC6343033; doi:10.1038/s41598-018-36551-5)
Supplement: Supplementary file 1 — Supplementary Information [file 41598_2018_36551_MOESM1_ESM.docx]

**Quantitative phase imaging reveals matrix stiffness-dependent growth and migration of cancer cells**

Yanfen Li^‡1^, Michael J Fanous^‡2^, Kristopher A. Kilian^*1,3^, Gabriel Popescu^*1,2^

1. Department of Bioengineering, University of Illinois at Urbana-Champaign, Urbana, Illinois 61801, USA

2. Quantitative Light Imaging Laboratory, Department of Electrical and Computer Engineering, Beckman Institute for Advanced Science and Technology, University of Illinois at Urbana-Champaign, Urbana, Illinois 61801, USA

3. School of Chemistry, School of Materials Science and Engineering, Australian Centre for Nanomedicine, University of New South Wales, Sydney, NSW 2052, Australia

‡These authors contributed equally to this work.

*To whom correspondence should be addressed: [k.kilian@unsw.edu.au](mailto:k.kilian@unsw.edu.au), [gpopescu@illinois.edu](mailto:gpopescu@illinois.edu)


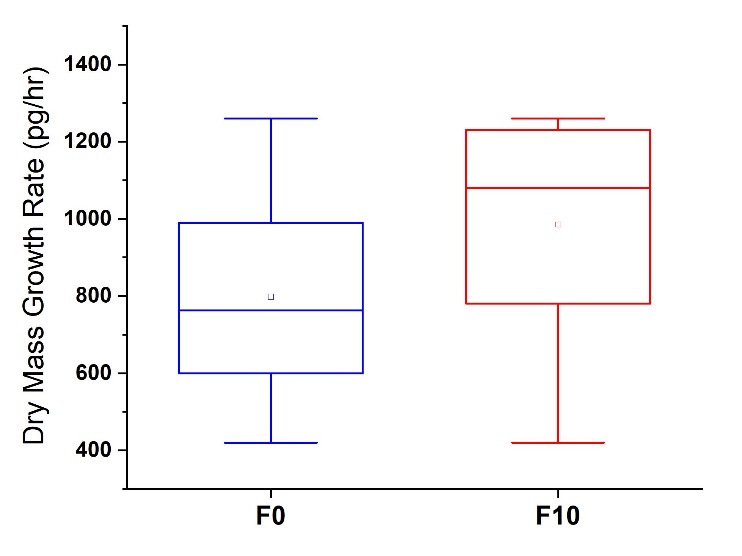


*

Supplemental Figure S1. Distribution of dry mass values comparing metastatic and non-metastatic growth for all substrates. (*) denotes a significant difference with p <0.05.

*

*


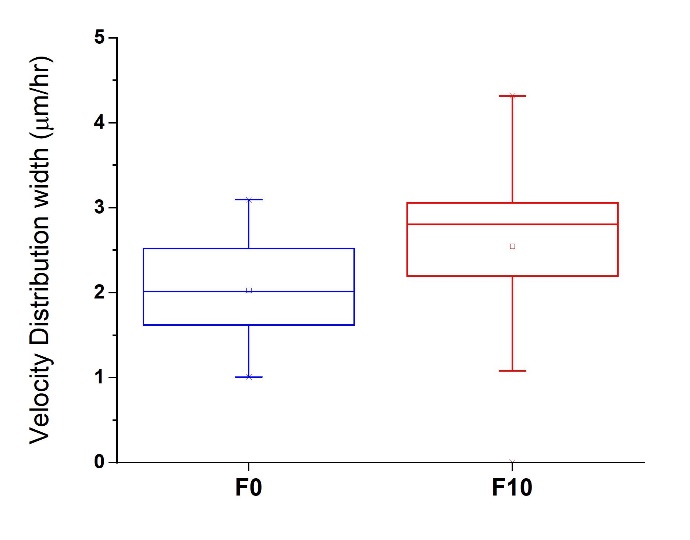


Supplemental Figure S2. Velocity width distribution for all categories of stiffness of metastatic and non-metastatic cells. (**) denotes a significant difference with p <0.01.


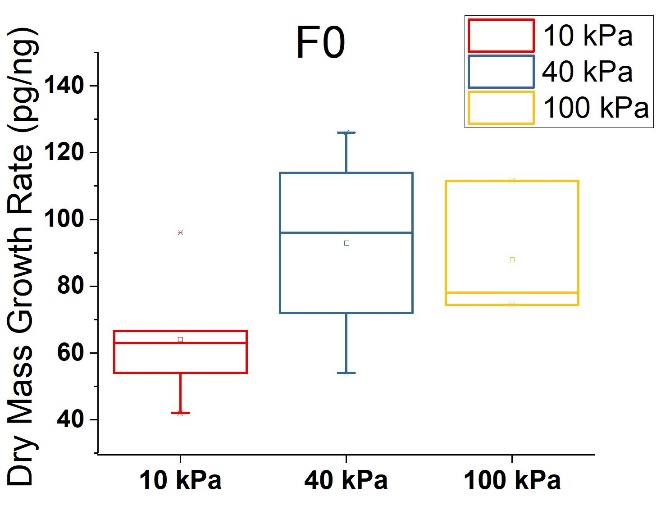


Supplemental Figure S3. Dry mass growth rate with respect to stiffness at a metastatic grade of F0.


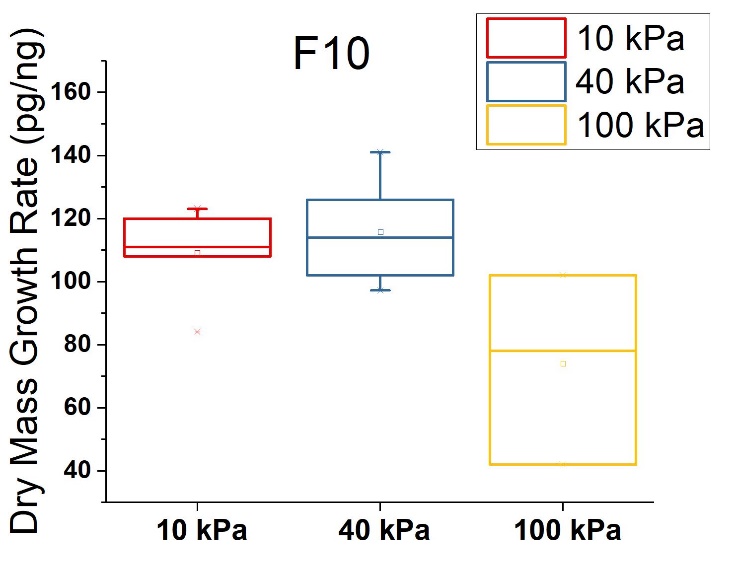


Supplemental Figure S4. Dry mass growth rate with respect to stiffness at a metastatic grade of F10.
